# Tell me who you’re coming with, I’ll tell you what you have!

**DOI:** 10.1192/j.eurpsy.2024.1703

**Published:** 2024-08-27

**Authors:** B. Castro Sousa, Z. Correia, J. Ramos

**Affiliations:** ^1^Chucb, ahflivoCahlivoC; ^2^Chucb, ahlivoC, Portugal

## Abstract

**Introduction:**

Psychiatric care is unique in its scope and complexity, as it involves the assessment and treatment of a wide variety of pathologies and, as these patients seek treatment, it is imperative to understand who accompanies them in clinical consultations and how the presence of these companions influences the treatment path. The dynamics between psychiatric patients and their companions in consultation, is extremely important as it can have significant implications for the effectiveness of treatment and the well-being of the patient.. Therefore, the presence of companions can take different forms, varying according to the diagnosis and needs of each person.

**Objectives:**

Thus, the authors intend, through carrying out a research study, to fill a critical gap in the understanding of presence of companions in psychiatric consultations, exploring the diversity of companions and their profiles in relation to patients psychiatric patients with specific diagnoses. Furthermore, they intend to understand how their presence impacts the process of adherence to the treatment.

**Methods:**

To achieve this, they defined a two-year follow-up period, where they examined in detail the composition of companions in psychiatric consultations, including who they are, their relationship with the patient and how this relationship varies according to different psychiatric diagnoses.

**Results:**

The presence of companions in psychiatric consultations is expected to prove to be a significant facet in the field of mental health, providing valuable insights into the dynamics of consultations and the treatment of patients with different psychiatric diagnoses. In this study we highlight how the presence of companions varied in relation to psychiatric diagnoses and how this influenced the process therapeutic. One of the main results was the identification of the different types of companions who were present at the consultations psychiatric disorders, reflecting the diversity of available social support and highlighting the importance of understanding the available support networks. A notable variation in the presence of companions in relation to psychiatric diagnoses was also observed, emphasizing the variations monitoring needs according to the nature of psychiatric disorders, suggesting the need for management strategies personalized treatment. This study also highlighted the influence of the presence of companions on doctor-patient communication and on adherence to treatment, in which the presence of family members often facilitated communication, allowing for a better understanding comprehensive history of the patient.

**Conclusions:**

In conclusion, this study contributes to a more holistic understanding of mental health care provision, highlighting the importance to consider not only the patient, but also the support context in which they are inserted

**Figure:**
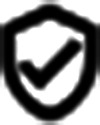

**Disclosure of Interest:**

None Declared

